# Cognitive-behavioral group therapy in major depressive disorder with focus on self-esteem and optimism: an interventional study

**DOI:** 10.1186/s12888-022-03918-y

**Published:** 2022-04-28

**Authors:** Radfar Moloud, Yavari Saeed, Haghighi Mahmonir, Gharaaghaji Asl Rasool

**Affiliations:** 1 Department of Psychiatric Nursing, School of Nursing and Midwifery, Urmia University of Medical Sciences, Pardis Nazlou. 11 Km of Nazlou Road, Urmia, Iran; 2 Department of Psychiatry, School of Medicine, Razi Hospital, Urmia University of Medical Sciences, Urmia, Iran; 3 Department of Community Medicine, School of Medicine, Urmia University of Medical Sciences, Urmia, Iran

**Keywords:** Cognitive behavioral therapy, Self-esteem, Optimism, Major depressive disorder

## Abstract

**Background:**

Major depressive disorder is a common psychological condition that can lead to negative individual and social consequences, the management of which is very important in treating the patients. The present study aimed to determine the effect of cognitive-behavioral group therapy on self-esteem and optimism in patients with major depressive disorder.

**Methods:**

This is a single-blinded, randomized controlled trial in which a total of 64 patients with major depressive disorder were recruited using convenience sampling and then randomly assigned to two groups of Cognitive-Behavioral Group Therapy (CBGT) and Treatment-As-Usual (TAU). Data collection tools consisted of a demographic questionnaire, the Rosenberg Self-Esteem Scale (RSES), and the Revised Life Orientation Test (LOT-R). In the pretest stage, participants in both groups completed the above questionnaires before the intervention. Patients in the CBGT group received eight 90-min sessions of cognitive-behavioral therapy during four weeks (two sessions a week). Then participants re-completed RSES and LOT-R immediately, three months, and six months after the intervention. Data were analyzed with SPSS software version 16.0 using chi-squared test, independent-samples t-test, and repeated measures Analysis of Variance. The significance level (*p*-value) was considered to be less than 0.05.

**Results:**

It was indicated that there was a statistically significant difference in the mean scores of self-esteem and optimism between the two groups immediately, three months, and six months after the intervention (*p* < *.05*). The mean scores of self-esteem and optimism in the CBGT group increased significantly after the intervention compared to before it, although these scores gradually decreased over the three measurement time points after the intervention.

**Conclusions:**

Based upon the results, it was concluded that the level of optimism and self-esteem increased significantly in the CBGT group after the intervention, although the levels of the above variables dropped again in the long run after the intervention due to the discontinuity of CBGT sessions. Therefore, it is necessary to take particular measures to regularly hold the sessions of CBGT for patients with major depressive disorder.

**Trial registration number:**

IRCT20140212016564N15, The date of registration: 20–09-2021, Retrospectively registered.

## Background

Major Depressive Disorder (MDD) is the most common recurrent mental health disorder [1]. Unlike the Diagnostic and Statistical Manual of Mental Disorders, fourth edition (DSM-IV), data on depressive disorders in DSM-5 are presented in a separate chapter due to its importance. All depression-related disorders have common particular characteristics including the presence of sadness, emptiness, or irritability along with cognitive and physical changes that significantly affect an individual's functional capacity [2]. MDD has a relatively chronic progression and high incidence and prevalence rates as well so that it is considered as one of the major health challenges worldwide [3]. Studies have reported an overall prevalence rate of 3–6% and the lifetime prevalence rate of 15–25% for MDD [4]. The incidence and prevalence rate of MDD has also grown significantly in Iran [5]. In a systematic review of 56 articles, the prevalence rate of depressive disorders in the Iranian population was reported to be 5.69–73% [6] and MDD was shown to be the most common psychiatric disorder with a prevalence rate of 12.7% [7].

Self-esteem is one of the concepts that is severely distorted in patients with depression [8]. Self-esteem refers to the belief that an individual has about his/her worth or value [9]. Poor self-esteem causes a number of psychological problems, such as anxiety, indifference, and feeling of loneliness in the depressed person that can lead to poor performance in adverse environmental conditions, high levels of stress, maladaptive responses, the persistence of depressive disorder, and suicide [10]. High self-esteem is an important part of self-concept and is also associated with adaptation [11]. Self-esteem is known as a predictor of depression [12] as some studies have shown that low self-esteem can lead to chronic depression. Some studies have also indicated that the level of self-esteem decreases as the level of depression increases [13]. Self-esteem has been conceptualized as both cause and effect of depression. The results of previous studies in this area have shown that there is a relationship between negative self-views and vulnerability to depression. [14]. People with low self-esteem have a strong tendency to gain excessive reassurance. Gaining excessive reassurance can lead to interpersonal rejection and further decline in the level of self-esteem, thereby increasing the risk of depression. [15].

Beck (1976) believes that three types of automatic negative thoughts including pessimistic attitudes towards oneself, the world, and the future lead to disease development in depressed people. This pessimism in depressed people undermines their self-esteem and sense of worth [16]. Optimism as the opposite of pessimism evokes positive emotions and improves the quality of coping with sources of stress and depression [17]. People with low levels of optimism have more dysfunctional or harmful expectations about their future, which in turn can lead to more severe depression symptoms [18]. In recent years, optimism has attracted the researchers' attention in many studies and has also been considered as one of the predictors of individuals' mental health [19]. An individual with an optimistic outlook on the future positively evaluates stressful situations and has a proper evaluation of their ability to get through problems [20]. Self-esteem and optimism help depressed people to resist the events that lead to failure and achieve more success in life than what others expect them [21]. The variables of optimism and self-esteem complement each other as positive psychological concepts. In other words, people with high levels of optimism have high levels of self-esteem. Regarding these results, a negative relationship is expected to be existed between the levels of optimism and self-esteem and the severity of depression. In addition, it can be stated that self-esteem can be a mediator between optimism and depression. Thus, there is a positive relationship between self-esteem and optimism and a negative relationship between self-esteem and depression. Besides, self-esteem and optimism can play a protective role on the symptoms of depression [22].

Cognitive Behavioral Therapy (CBT) is a form of speech therapy that helps manage problems by altering a person's thoughts and behaviors. Moreover, CBT is a commonly used treatment method for depression [23]. This method is based upon the continuous connection of the content of a person's thoughts, feelings, physical conditions, and performance [24]. Regarding that thoughts and actions often occur simultaneously, behavioral and cognitive techniques are used interchangeably for patients so that the term CBT is used to emphasize the close correlation between them [25].

Cognitive-Behavioral Group Therapy (CBGT) is a more cost-effective treatment method in depressed patients compared to the individual CBT since people can better express their thoughts and feelings in the group and have the opportunity to have an interpersonal discussion, cooperation, and participation [26]. Researches on this treatment method have shown different results. Jiang et al. (2018) found that CBT has positive effects on quality of life, self-esteem, and mood in patients with heart failure [27]. Saeidi et al. (2015) showed that an optimism training program is effective in improving depression and life satisfaction in women on the verge of divorce [28]. On the other hand, Jannati et al. (2017) indicated that the CBGT has no effect on self-esteem in patients with bipolar I disorder [29].

In Iran, pharmacotherapy and electroconvulsive therapy are known as the most common treatment method for psychiatric disorders in both acute and chronic stages [30]. Regarding that the non-pharmacological treatment methods have no place in the routine care programs of the hospitals, patients can not enjoy the advantages of psychological therapies e.g. CBT. This is while some of the depression symptoms seem to respond better to these types of treatment.

According to what has been said, levels of self-esteem and optimism decrease in patients with depression and the modulation of these negative complications can be effective in improving individual and social functioning, quality of life, and ultimately the treatment process in these patients. Therefore, considering that psychological therapies do not have the side effects of pharmacotherapy, the present study was conducted to determine the effect of cognitive-behavioral group therapy on self-esteem and optimism in patients with major depressive disorder.

## Methods

### Participants & Study Setting

The aim of the study is to determine the effect of cognitive-behavioral group therapy on self-esteem and optimism in patients with major depressive disorder.

Prior to the beginning of the study, ethical approval was received from the Research Ethics Committee of Urmia University of Medical Sciences (Ethics No. IR.UMSU.REC.1397.116). A single-blinded, parallel, randomized controlled trial was conducted from September 2018 to March 2019 at Razi Psychiatric Hospital, which is the only psychiatric medical center in Urmia. The target population for this study consisted of outpatients with MDD referred to the above-mentioned hospital.

Inclusion criteria consisted of the followings: (a) exact diagnosis of MDD based on the DSM-5 diagnostic criteria and a diagnostic clinical interview, (b) granting informed consent to participate in the study, (c) receiving no psychotherapy services and counseling during the CBGT sessions, (d) having no history of participation in similar studies, (e) having at least primary education, and (f) having no substance abuse. On the other hand, exclusion criteria included the followings: (a) being absent for more than two sessions, (b) administration of electroconvulsive therapy, and (c) substance abuse during the intervention.

Sampling was conducted in morning shifts (from 8 a.m. to 2 p.m.) on working days so that it lasted for 32 days. A number of 154 patients with MDD were evaluated by the researchers, out of which 121 met the inclusion criteria. Out of 121 eligible patients, 24 were reluctant to attend the CBGT sessions due to residency problems and 17 withdrew from the study before the beginning of the intervention. Ultimately, a total of 80 patients entered the study and were assigned to two groups of CBGT and Treatment-As-Usual (TAU) using random allocation. For this purpose, each of the letters A (CBT) and B (TAU) was written on 40 cards. Then each patient randomly selected one of the cards and was allocated to the CBGT or the TAU group based on the letter written on the card.

### Intervention

The CBGT sessions were held by the second author (Master's student in psychiatric nursing) under the supervision of the first (doctoral in psychiatric nursing) and the third author (psychiatrist).

In a briefing session, the study objective was explained to the patients in the TAU group and then written informed consent was obtained from them. At the end of this session, patients in this group completed three questionnaires and they were also requested to refer to the researcher for re-completing the questionnaires at three specified time-points. During the study process, the TAU group received routine treatments including psychiatrist visit, pharmacotherapy and psychoedjucation.

**Fig. 1:**
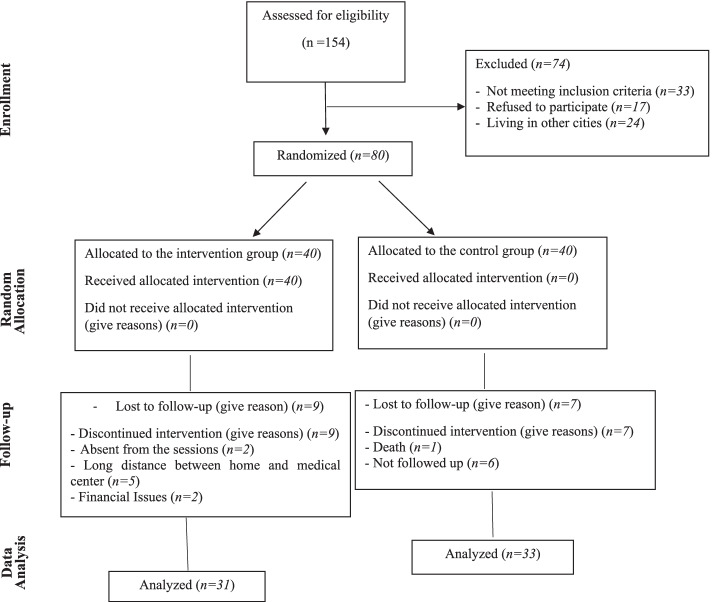
Flow chart of patient inclusion, follow-up, and data analysis

In a briefing session, written informed consent was also received from patients in the CBGT group after providing them with necessary explanations on the study objective. In this session, patients in this group were randomly divided into 4 groups of 10 patients and then completed the questionnaires. Moreover, the holding time of the next sessions was determined for each group at the end of the first session. In addition to the routine treatment program, four groups of intervention received eight sessions of CBGT during four weeks (two sessions a week). It should be noted that each session lasted for 60–90 min. The content of the sessions was prepared based on the views of Beck (1979) [31] and Rusello and Bernal (2007) [32] (Table 1). Each session was held in accordance with the following stages: (1) reviewing the content of the previous session, (2) presenting a new topic, (3) group discussion about the new topic, (4) summarizing the content with patients and catering. The sessions were also held in a quiet, comfortable, and distraction-free environment. According to the principles of CBGT, the chairs in the discussion room were arranged in a circle so that the members had face-to-face communication with each other and none of them felt superior to others. In all sessions, the researcher was the group leader. During the sessions, 9 patients in the CBGT group were excluded from the study due to absenteeism, residency, and financial issues. Besides, 7 patients in the TAU group were excluded from the study due to death and lack of referral to the researcher. Finally, a total of 64 patients (*n* = *31* in the CBGT group, *n* = *33* in the TAU group) remained in the study. At the post-test stage, patients in both groups completed the questionnaires immediately, three months, and six months after the intervention. At this stage, patients completed the questionnaires when they referred to the physician for monthly visits (Fig. 1). In order to comply with the research ethics, a treatment session was held for the patients in the TAU group after the completion of the last questionnaire to boost their levels of self-esteem and optimism.

**Table 1:** The content of the CBGT sessions

Session No	Treatment Content
1^st^	- Introducing the CBT therapist, getting patients acquainted with each other, and establishing a trust-based relationship - Expressing the importance and purpose of both the formation of a group and the provision of information on CBT - Providing general information (specification of time, place, length, and number of the sessions) - Setting rules and regulations for the group members (attending the session on time, respecting other patients when talking, and leaving the group in case of absence for more than two sessions) - Conducting pre-test and making a summary of the whole session
2^nd^	- Encouraging patients to express their thoughts, attitudes, and experiences about depression - Presenting the content remained by the researcher - Summarizing the content of the session and giving patients assignment (identification and preparation of a list of patient's depression symptoms)
3^rd^	- Reviewing the assignments given in the previous session - Learning how to deal with the problems and the ups and downs of life by talking and sharing ideas about having a rational, healthy, purposeful, and flexible life - Talking about one's thoughts and beliefs - Accepting that beliefs can be changed - Relationship of thoughts and beliefs with psycho-emotional reactions and behavior Summarizing the content of the session and giving patients assignments (identification and preparation of a list of patients' negative and positive beliefs and thoughts)
4^th^	- Reviewing the assignments given in the previous session - Talking to group members about self-esteem and its importance - Identification of the characteristics of people with high levels of self-esteem and finding the subjects with these characteristics - Making patients familiar with the barriers to self-esteem caused by cognitive distortions (e.g. considering oneself as an individual with poor self-esteemed) - Expressing effective ways to overcome barriers to self-esteem - Summarizing the content of the session and giving patients assignments (identification and preparation of a list of barriers to self-esteem and provision of solutions for it)
5^th^	- Reviewing the assignments given in the previous session - Talking about the concept of self-acceptance and related social skills - Identification and enhancement of the core strengths, feelings, and positive emotions - Summarizing the content of the session and giving patients assignments for the next session (identification and preparation of a list of irrational thoughts and beliefs in oneself, identification of one's strengths and weaknesses)
6^th^	- Reviewing the assignments given in the previous session - Talking about optimism and its importance - Identification of the characteristics of optimists and finding the subjects with these characteristics - Making patients familiar with the barriers to optimism resulted from cognitive distortions - Presenting effective ways to overcome barriers to positive thoughts - Summarizing the content of the session and giving patients assignments (identification and preparation of a list of the traits of optimistic people)
7^th^	- Reviewing the assignments given in the previous session - Enumerating the blessings (considering the little blessings of life and write down 3 of them every day) - Providing information on how to manage negative behavior (having a clear plan to reduce negative behaviors) - Conducting cognitive interventions (correction of distorted and dysfunctional thoughts as well as the disease-related beliefs that can affect self-esteem and optimism) - Dealing with negative thoughts and feelings through identification of skills, positive thoughts, and feelings that improve optimism and self-esteem - Summarizing the content of the session and giving patients assignments (preparation of a plan for boosting self-esteem and optimism and overcoming barriers)
8^th^	- Reviewing the assignments given in the previous session - Getting feedback from patients about the treatment plan - Maintaining therapeutic effects (getting feedback from subjects and providing practices for future use) - Providing an opportunity to end the CBGT sessions - Conducting post-test and acknowledgment

### Data Collection Tools

In this study, data were collected using a demographic questionnaire, the Rosenberg Self-Esteem Scale (RSES), and the Revised Life Orientation Test (LOT-R).

The demographic questionnaire included items on age, gender, marital status, level of education, occupation, history of physical illness, drug history, duration of present illness, number of hospitalizations).

### The Rosenberg Self-Esteem Scale (RSES)

The RSES is a reliable and valid self-report scale for measuring the level of self-esteem, which was first developed by Rosenberg in 1965. This scale is one of the most widely used tools to assess self-esteem and contains 10 statements all of which are scored on a 4-point Likert scale. It should be stated that items 1 to 5 are scored positively (Strongly Agree = 3 to Strongly Disagree = 0) and items 6 to 10 are scored reversely (Strongly Agree = 0 to Strongly Disagree = 3). The overall score of the scale ranges from 0 to 30 so that the higher score indicates a higher level of self-esteem [33]. In psychometrics of this scale, Rosenberg (2015) reported the correlation coefficient between the individual and the collective self-esteem to be *r* = *0.34*. He also reported Cronbach's alpha and test–retest reliability to be *α* = *0.93* and *r* = *0.85*, respectively [34]. In the psychometrics of the Farsi version of the RSES, the test–retest reliability coefficient at an interval of three weeks (*n* = *29*) was obtained 0.84 and the internal consistency was assessed using Cronbach's alpha coefficient, which turned out to be *α* = *0.83* [35].

### The Revised Life Orientation Test (LOT-R)

The LOT-R is a 10-item standard instrument for assessing one's level of optimism. All items of this instrument are scored on a 5-point Likert scale from strongly agree = 0 to strongly disagree = 4. In this scale, items 1, 4, and 10 are scored positively, while items 3, 7, and 9 are scored reversely in valence. The overall score of this instrument ranges between 0 and 24 so a lower score indicates a lower level of optimism. Based upon the confirmatory factor analysis of LOT-R, the correlation coefficient between factor loadings was reported to be *0.59-0.80*. Moreover, Cronbach's alpha for Lot-R was obtained to be *α* = *0.71*[36]. In Iran, the reliability of this instrument was calculated using Cronbach's alpha and test–retest reliability, each of which was reported to be *α* = *0.74* and *r* = *0.87*, respectively. The concurrent validity coefficient between the optimism and two factors of depression and self-mastery was worked out to be *ρ* = *-0.64* and *ρ* = *0.72*, respectively [37].

### Data Analysis

Data were first entered into SPSS Statistics for Windows, version 16.0 (SPSS Inc., Chicago, Ill., USA). The clinical and demographic characteristics of the two groups were compared using the chi-squared test and independent-samples t-test. To compare the mean scores of self-esteem and optimism between the two groups at four time points, the independent-samples t-test was utilized. For this purpose, Cohen's d index was determined by calculating the mean difference between the two groups and dividing it by the pooled Standard Deviation (SD).

Cohen's d = (M2 − M1) / SD pooled, SD pooled = √ ((SD_1_^2^ + SD_2_^2^) / 2).

To evaluate the significance of the difference between the mean scores of self-esteem and optimism at four time points in each group, repeated-measures Analysis of Variance (rANOVA) was used. Before conducting the rANOVA, the condition of equality of between-groups variances was met by observing the assumptions and performing the necessary tests. In this regard, it should be mentioned that the amount of residual variance of the dependent variables was equal in all groups. Furthermore, the significance level (*p*-value) was considered to be less than 0.05. All analyses were performed by a researcher who was blind to the data.

## Results

The findings of the present study were derived from the analysis of data obtained from 64 patients (*n* = *33* in the TAU group and *n* = *31* in the CBT group). In this study, there was 20% of sample attrition so that a number of 7 and 9 patients were excluded from the TAU and the CBGT group, respectively. This sample attrition caused no significant difference in the demographic characteristics of the two groups. The clinical and demographic characteristics of participants are presented in Table 2.

**Table 2:** Clinical and demographic characteristics of the cognitive behavior group therapy (CBGT) and treatment as usual (TAU) groups

Qualitative Variable		CBGT group (*N* = 31) N (%)	TAU group (*N* = 33) N (%)	test
Gender	Male	14 (45.2%)	16 (46.9%)	*P* = *0.890*
	Female	17 (54.8%)	17 (53.1%)	
marital status	Married	16 (51.6%)	26 (78.8%)	*P* = *0.066*
	Single	8 (25.8%)	4 (12.1%)	
	Widow	7 (22.6%)	3 (9.1%)	
Educational status	Secondary education	28 (90.3%)	32 (97%)	*P* = *0.347*
	High education	3 (9.7%)	1 (30%)	
Occupational	Employed	7 (22.6%)	12 (36.4%)	*P* = *0.280*
	Unemployed	24 (77.4%)	21 (63.6%)	
History of previous illness	Yes	3 (9.7%0	5 (15.2%)	*P* = *0.709*
	No	28 (90.3%)	28 (84.8%)	
Quantitative Variable		CBGT group (N = 31) Mean (SD)	TAU group (N = 33) Mean (SD)	Independent t-test
Age (year)		37.26 (9.40)	42.21 (10.19)	*P* = *0.051*
Duration of Diagnosis		6.73 (5.35)	5.56 (3.87)	*P* = *0.340*
Frequency of hospitalizations		1.82 (1.11)	2.20 (1.79)	*P* = *0.410*

Based on the results, there was no statistically significant difference between the two groups in terms of demographic characteristics. In other words, the TAU and CBGT groups were homogenous in this regard. Moreover, both groups were matched in terms of drug history (type and frequency of medications used). Patients with MDD in this study were treated with SSRI antidepressants (include: fluoxetine, sertraline, citalopram, escitalopram) or TCA (include: nortriptyline, maprotiline, doxepin) or SNRI (include: venlafaxine) with or without a benzodiazepine (include: clonazepam, lorazepam, alprazolam) to treat insomnia.

The mean scores of self-esteem and optimism at four measurement time points are presented in Table 3. The results of data analysis showed that there was no statistically significant difference in the mean scores of self-esteem and optimism between the two groups before the intervention. However, this difference was found to be statistically significant between the two groups at three time points of immediately, three months, and six months after the intervention so that the Cohen's d at all three time points was more than 0.7, which showed a large effect size in this regard. The results of rANOVA on the significance of the difference in the mean scores of self-esteem and optimism in the two groups are presented in Table 4. In this table, three types of interaction effects are also reviewed. Based on the results, the time by intervention interaction was found to be significant (*p ˂ 0.001*). This indicated that there was a significant difference in the mean scores of self-esteem and optimism between the two groups at different time points. In other words, the trend of the mean scores of response variables was not similar in the two groups over time.

**Table 3:** Means and standard deviations (SD) on the Self-steam and Optimism baseline and follow-up (measurement for four time points)

	Base line	Completion of CBGT	3 month after CBGT	6 month after CBGT
	CBGT mean TAU mean (SD) (SD)	CBGT mean TAU mean (SD) (SD) Cohen’s d	CBGT mean TAU mean (SD) (SD) Cohen’s d	CBGT mean TAU mean (SD) (SD) Cohen’s d
Self-steam	14.68 (1.30) 14.33 (1.40) *P* = *0.315*	16.84 (2.03) 14.67 (1.97) *P* = *0.001* 1.08	16.19(2.12) 14.00 (1.43) *P* = *0.001* 1.21	15.52(1.74) 13.88(1.08) *P* = *0.001* 1.32
Optimism	9.90 (1.68) 9.91 (1.92) *P* = *0.990*	12.85 (1.46) 10.33(1.45) *P* = *0.001* 1.73	12.60 (1.84) 10.64 (1.70) *P* = *0.001* 1.10	12.30 (1.03) 10.23 (1.50) *P* = *0.001* 1.60

**Table 4:** The results of the repeated measure ANOVA in comparison of the mean scores of self-esteem and optimism at three time points of immediately, 3 months, and 6 months after the intervention in the CBGT and TAU groups

**Variable**	**Interaction effect**	***SST***^a^	***DF***^b^	***MS***^*c*^	***F***	***p*-value**	***η* (eta)**
**Self-esteem**	Time	*47.645*	*3*	*18.882*	*6.616*	0	*.099*
	Group by Time	*40.742*	*3*	*13.581*	*5.657*	*.001*	*.086*
	Group	*154.903*	*1*	*154.903*	*37.098*	*.001*	*.382*
	Error	*250.532*	*60*	*4.176*			
**Optimism**	Time	*87.86*	*3*	*29.28*	*14.09*	*.001*	*.227*
	Group by Time	*41.18*	*3*	*13.72*	*6.60*	*.001*	*.121*
	Group	*137.81*	*1*	*137.81*	*31.54*	*.001*	*.397*
	Error	*209.69*	*48*	*4.36*			

^a^Sum of Squares

^b^Degree of Freedom

^c^Mean of Squares

In this study, the main effect of time was also found to be significant and this showed a statistically significant difference between the mean scores of self-esteem and optimism at different time points in the CBGT group (*p ˂ 0.001*). The main effect of the intervention also revealed that there was a statistically significant difference in the mean scores of self-esteem and optimism between the two groups regardless of the effect of time. In other words, it was concluded that the mean scores of self-esteem and optimism in the TAU group were significantly lower than in the CBGT group (*p ˂ 0.001*). Considering the partial Eta squared, the effect of intervention had the highest interaction effect on both variables of self-esteem (*η2* = *0.382)* and optimism (*η2* = *0.397*).

**Fig. 2:**
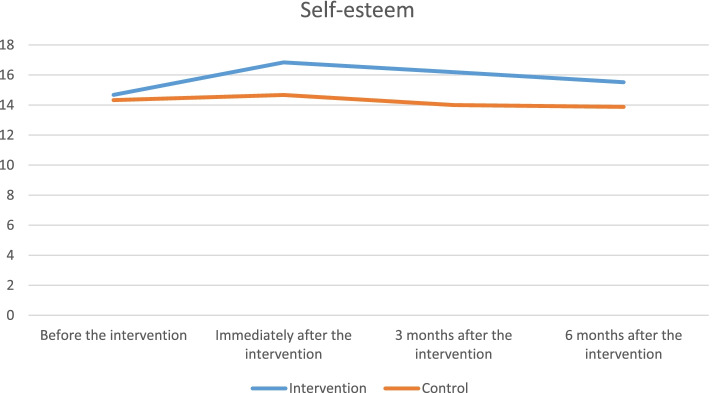
Changes in the mean score of self-esteem in two groups throughout the measurement time points

**Fig. 3:**
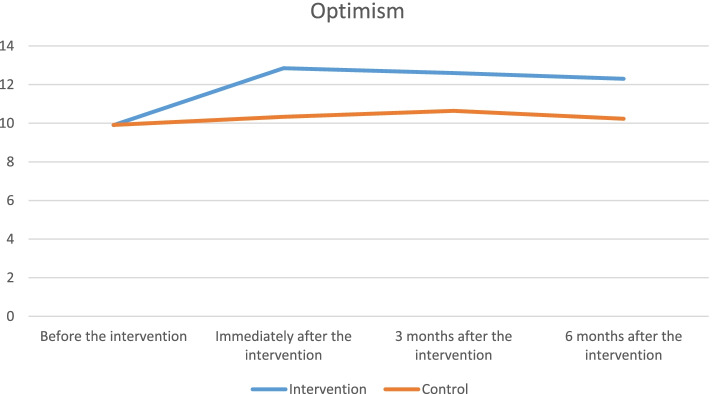
Changes in the mean score of optimism in two groups throughout the measurement time points

The changes in the mean scores of self-esteem and optimism at different time points in the two groups are provided in Figs. 2 and 3. Based on the results, it was indicated that the mean scores of self-esteem and optimism significantly increased in the CBGT group at time points of immediately, three months, and six months after the intervention compared to before it. Moreover, the highest mean score was obtained at the measurement time point of immediately after the intervention, while the overall mean score gradually decreased over time (three and six months after the intervention). In contrast, differences in the mean score of self-esteem and optimism in the TAU group did not found to be significant at the four measurement time points.

## Discussion

This study aimed to investigate the effect of CBGT on self-esteem and optimism in patients with MDD. Based upon the finding derived from data analysis, it was shown that the mean scores of self-esteem and optimism increased significantly in the CBGT group. Despite the fact that the results showed a significant increase at all three follow-up time points compared to before the intervention, the mean scores of both response variables gradually decreased over time. Therefore, a continuous and dynamic CBGT can improve the level of self-esteem and optimism in patients with MDD. Many studies have examined the effectiveness of CBT [38–47]. In these studies, the researchers evaluated the primary outcomes include psychological wellbeing [43–47], depression symptom [45, 46, 48], mood and anxiety symptom [39, 46, 47, 49], depression [38, 47, 49], self-stigma [46], quality of life [45], self-esteem [40–45, 47, 49], optimization, social support[43] and the secondary outcomes of CBT include self-esteem [48], automatic thoughts [38] in different samples; university student [43], obese females [42], physically disabled females [44], early psychosis patients [48], patients with depression [38, 46], patients with bipolar disorder [39], psychiatric patients [50], patients with MDD [45], people with a learning disability [40]. In most of which indicated that CBT has improved self-esteem after the intervention. [38–41, 43, 44, 46–48, 50]. A number of three studies have examined the effect of CBT on self-esteem in depressed patients. [42, 45, 49]. One of which was a case report on a patient with symptoms of depression, anxiety, and low self-esteem. In this study, after conducting 12 sessions of CBT in a six-month period and having a one-year follow-up, a greater effect size was reported for symptoms of depression, anxiety, and self-esteem [49]. Another study was a meta-analysis on the efficacy of CBT in different mental health disorders. In this meta-analysis, researchers reported that the studies on patients with depression had different results as some of them have shown high efficacy of CBT and some others have shown poor efficacy of this treatment method [50]. In another study, the researchers examined the effect of CBT in the early phase of psychosis with a focus on depression and low self-esteem in two groups of intervention and control. The results of this study concealed that the level of depression and self-esteem improved in both groups during treatment and follow-up, although CBT was not found to be more advantageous than conventional treatment methods [48]. This result is not consistent with the results of our study. This inconsistency between the results may be rooted in differences in routine treatment programs. In the above study, the main components of routine treatment included pharmacotherapy, regular psychiatric evaluation, and continuous follow-up. Moreover, some patients received community-based mental health services and most of them received psychotherapy, especially cognitive therapy. This is while the routine treatments in our study only included pharmacotherapy and monthly psychiatrist's visit. In our study, CBGT seemed to have a positive effect on the level of self-esteem since the subjects did not normally benefit from psychological therapies.

CBT is a purposeful effort to maintain the positive effects of behavioral therapy and integrate cognitive activities for bringing about therapeutic changes [51]. This therapeutic approach emphasizes the important role of cognition in the occurrence of behavioral and emotional changes [24, 31]. CBT is an organized and short-term problem-oriented treatment that aims to modify incorrect and irrational cognitions [23, 52]. This treatment method also helps the patient to assess and control his/her negative thoughts. This ability to control negative thoughts constitutes the core of CBT. CBT is largely based on self-help so that the therapist's goal is to help the patient develop the necessary skills in solving not only the current problems but also similar problems in the future [53]. In our study, one of the female patients described herself in the initial sessions as follows: *"I do not consider myself as an important individual and those who have high self-esteem are successful."* During subsequent CBGT sessions, she changed her mind as follows: *"I came to the conclusion that the position I had considered for myself did not deserve me at all and was inferior to that I really deserve."*

Based on the results of our study, the mean score of optimism increased significantly at all three follow-up time points compared to before the intervention. In studies on the effect of CBT on optimism in patients with depression, optimism has been evaluated not as a single variable but as a component of positive mental health well-being [54–57] In line with the results of our study, the results of the above studies indicated the positive effect of CBT on optimism. In addition, the results of the studies with the research population other than patients with depression also demonstrated the positive effect of CBT and other psychological interventions on the level of optimism [58, 59]. In line with the results of our study, Bolier et al. (2013) [54] reported that there was a less but still significant increase in the mean score of optimism at two follow-up time points of three and six months after the intervention. Therefore, it can be stated that the effect of the intervention is maintained to some extent over time. However, CBT should be conducted continuously to produce a stable effect in this regard.

Seligman et al. (2016) state that optimism can be learned and we are optimistic if we learn to have an external, specific, and temporary attributional style in the face of unpleasant events and a general and permanent internal attributional style in the face of pleasant events. They also found that training on how to change attributional styles reduced the levels of depression and anxiety in individuals [60]. According to the definition of optimism, human actions are greatly influenced by the expectations of the results of those actions. Optimists have positive future expectations [61].

In our study, one of the female patients expressed her opinion on optimism in the initial sessions as follows: *"I am not very optimistic because the future and future events do not appear meaningful and clear and these all seem absurd to me."* However, after the completion of the sessions, her opinion changed as follows: *"I am a little more optimistic about the future now and I believe that negative things are not always going to happen."*

## Study Strengths & Limitations

This study had several strengths, one of which was the consideration of four measurement time points (one baseline and three follow-ups). Another strength was the diagnosis of depression in samples based on diagnostic interviews and DSM-V criteria.

On the other hand, this study had limitations, the first of which was the refusal of some patients to attend the CBGT sessions due to the long distance between their residences and the hospital. Second, the patients' level of depression has not been measured after intervention and this can be considered as one of the limitations of study.

## Conclusion

Based on the findings of the present study, it was concluded that showed that CBGT is an effective treatment method for improving the levels of self-esteem and optimism in patients with MDD, although its positive effects gradually decreased with the termination of the sessions. Therefore, in order to continue the effects of CBGT, it is necessary to hold regular and continuous treatment sessions alongside pharmacotherapy for these patients. This combination therapy can change the way of thinking and improve communication in these patients by promoting their levels of self-esteem and optimism.

## Data Availability

The datasets used and/or analyzed during the current study are available from the corresponding author on reasonable request.

## Abbreviations

CBT: Cognitive-Behavioral Therapy
CBGT: Cognitive-Behavioral Group Therapy
TAU: Treatment-As-Usual
RSES: Rosenberg Self-Esteem Scale
LOT-R: Revised Life Orientation Test
MDD: Major Depressive Disorder
DSM-IV: Diagnostic and Statistical Manual of Mental Disorders, fourth edition
DSM-5: Diagnostic and Statistical Manual of Mental Disorders, fifth edition
